# Associations Between Nursing Faculty Expertise in the United Nations Sustainable Development Goals and Research Impact Metrics: A Cross‐Sectional Study

**DOI:** 10.1155/jonm/9740644

**Published:** 2026-04-07

**Authors:** Suebsarn Ruksakulpiwat, Witchuda Thongking, Atsadaporn Niyomyart, Chitchanok Benjasirisan, Lalipat Phianhasin, Ruttanaporn Kongkar, Nattaya Praha, Jon Adams, Austen El-Osta

**Affiliations:** ^1^ Department of Medical Nursing, Faculty of Nursing, Mahidol University, Bangkok, Thailand, mahidol.ac.th; ^2^ Department of Control Systems and Instrumentation Engineering, King Mongkut’s University of Technology Thonburi, Bangkok, Thailand, kmutt.ac.th; ^3^ Ramathibodi School of Nursing, Faculty of Medicine, Ramathibodi Hospital, Mahidol University, Bangkok, Thailand, mahidol.ac.th; ^4^ School of Public Health, The University of Technology Sydney, Sydney, Australia, uts.edu.au; ^5^ Self-Care Academic Research Unit (SCARU), Primary Care & Public Health, School of Public Health, Imperial College London, London, UK, imperial.ac.uk

**Keywords:** machine learning, nursing faculty, research impact metrics, UN SDGs, XGBoost

## Abstract

**Background:**

The United Nations Sustainable Development Goals (SDGs) offer a comprehensive global framework for promoting health, equity, and sustainability. Whereas alignment with the SDGs is increasingly encouraged in academic institutions, the extent to which faculty expertise in SDGs influences traditional research impact metrics remains insufficiently explored.

**Objective:**

To investigate the relationship between nursing faculty expertise in SDGs and research impact metrics.

**Methods:**

A retrospective cross‐sectional design was employed using data from 121 nursing faculty members at Mahidol University, Thailand. Information on SDG‐related expertise and research performance was obtained from the Mahidol University Research Excellence Database (MUREX) and Scopus. SDG expertise was operationalized using SDG alignment data derived from the Scopus Author Profile, which applies machine learning and keyword‐based text mining to map publications to the 17 SDGs. Descriptive statistics, Pearson’s correlation, and multiple linear regression analyses were used to examine associations between SDG expertise, academic experience, and research impact metrics, including H‐index, citation count, and research output. Extreme Gradient Boosting (XGBoost) was applied as a complementary machine learning approach to identify influential features and potential nonlinear patterns, with the Synthetic Minority Oversampling Technique (SMOTE) used to address imbalance in categorical SDG expertise classes.

**Results:**

Faculty members with greater expertise in SDGs demonstrated significantly higher research impact metrics. SDG expertise significantly predicted H‐index (*β* = 0.65, *p* < 0.001), total citations (*β* = 31.78, *p* = 0.004), and total research output (*β* = 2.41, *p* < 0.001). Research experience was also a significant predictor of research impact. Machine learning analyses identified SDG expertise breadth and international collaboration as influential features, and faculty aligned with SDG13 (Climate Action) demonstrated a higher proportion of top‐cited publications.

**Conclusion:**

SDG expertise is a key determinant of academic impact, reinforcing the need for greater institutional support for SDG‐aligned research. Interdisciplinary collaboration and engagement with broader sustainability challenges may enhance faculty research visibility. Future research should explore longitudinal trends and policy implications for integrating SDGs into faculty assessment frameworks.

## 1. Background

The United Nations (UN) Sustainable Development Goals (SDGs), launched in 2015, represent a global commitment to addressing a broad spectrum of critical issues, including health, inequality, climate change, and sustainable development [1]. The SDGs aim to promote international cooperation and comprehensively mobilize resources to tackle these challenges. Specifically, SDG3 (Good Health and Well‐being) [2] and SDG17 (Partnerships for the Goals) [3] are highly relevant to the field of nursing. Whereas SDG3 seeks to ensure healthy lives and promote well‐being for all, a mission central to nursing practice [2], SDG17 emphasizes the importance of partnerships and collaborations to achieve the SDGs, highlighting the need for collective efforts in research and practice [3].

Nursing faculty plays a crucial role in advancing research that supports these global goals by influencing health policies, informing clinical practice, and contributing to evidence‐based care [4, 5].

Research impact metrics are vital for assessing the reach and effectiveness of academic contributions [6, 7]. Expertise in SDGs might influence these metrics by directing research agendas toward areas of high relevance and global importance. For instance, a faculty with expertise in SDG3 will likely focus on research addressing major health issues, potentially leading to higher citations and greater visibility in their field [8]. Similarly, expertise in SDG17 may promote collaborations and partnerships that enhance research output and impact [5].

Recent evidence also demonstrates the value of machine learning for modeling SDG‐related outcomes. For example, Chenary et al. used machine learning approaches to forecast SDG scores through 2030, highlighting the potential of computational models to support SDG‐oriented evaluation and decision‐making [9]. This evidence supports the relevance of incorporating machine learning alongside traditional statistical analyses when examining SDG‐related phenomena, particularly where relationships may be complex or non‐linear.

Existing research has not systematically examined how the nursing faculty’s expertise in SDGs influences key research impact metrics (e.g., H‐index and total citations). Most prior SDG‐related studies have focused on national or regional progress, policy evaluation, or forecasting aggregate SDG performance using statistical or machine learning approaches [9] rather than examining SDG expertise at the individual faculty level within academic institutions. In particular, empirical evidence linking faculty‐level SDG expertise to traditional bibliometric indicators of research impact in nursing remains limited.

Understanding these relationships is essential for informing research strategies, funding allocation, and policy decisions in higher education. Accordingly, this study examines nursing faculty characteristics and investigates associations between faculty expertise in the UN SDGs and research impact metrics, providing insight into how alignment with global health priorities may enhance academic visibility and influence.

## 2. Materials and Methods

### 2.1. Study Design and Setting

This study employed a retrospective cross‐sectional design to examine the relationship between nursing faculty expertise in the UN SDGs and research impact metrics. The analysis focused on nursing faculty members at Mahidol University, Thailand, a premier academic institution in the region recognized for its research excellence and international collaborations. Data were retrospectively extracted from publicly accessible sources, including the Mahidol University Research Excellence Database (MUREX) and Scopus [10, 11]. For each faculty member, all available publications and research performance data were collected from the year of their first Scopus‐indexed publication through 2024.

MUREX functions as a central hub for showcasing research achievements and plays a crucial role in administrative reporting. The portal, which can be accessed through a dedicated website [10], provides public access to a wide range of research outputs, including activities, events, projects, recognitions, and media interactions, while also tracking research impact and promoting global partnerships. Data in the system are also optimized for easy discovery and indexing by search engines [10]. Additional data were obtained from the Scopus database, which offered unique profiles for each researcher, documenting their publication activity based on peer‐reviewed articles and other indexed sources in Scopus [11].

### 2.2. Participants

Participants were included if they were faculty members (i) affiliated with the School or Faculty of Nursing at Mahidol University, (ii) held an academic title of Lecturer, Assistant Professor, Associate Professor, or Full Professor, and (iii) had at least one research publication indexed in Scopus. Faculty members without Scopus‐indexed publications and faculty members with administrative roles but no active research output were excluded. A total of 121 nursing faculty members met the eligibility criteria and were included in the study.

### 2.3. Variables and Data Sources

Data were preliminary extracted from MUREX and Scopus, two comprehensive academic databases tracking research activities and impact metrics [10, 11]. The variables identified and analyzed, along with their operational definitions, in this study are presented in Table 1.

**TABLE 1:** Variables and their definitions.

Variable	Operational definition
Academic title	Refers to the current academic title that the faculty member is holding, including “Lecturer,” “Assistant Professor,” “Associate Professor,” or “Full Professor.”
Expertise related to UN SDGs	The UN SDGs (Supporting Data 1) were used as a framework to determine the nursing research expertise of each participant. SDG alignment data were obtained from the Scopus Author Profile, which applies an SDG classification methodology to map published documents to the 17 SDGs. This mapping framework uses a combination of machine learning techniques and keyword‐based text mining applied to publication titles, abstracts, and indexed keywords to assign SDG relevance to individual documents. The number of documents attributed to each SDG for a given author was extracted directly from Scopus and cross‐referenced within the MUREX portal where applicable. To ensure the accuracy and consistency of these classifications, our research team independently reviewed the assigned SDG tags for each faculty member. This verification process involved cross‐checking the algorithm‐generated classifications with the individual research outputs’ content and thematic focus. Discrepancies were resolved through team consensus to maintain the integrity of the data used in the analysis.
Year of research experience	The number of years of research experience was calculated from the publication of the first research paper indexed in a Scopus journal until 2024, with each year counted as one full year. For example, if “Researcher A” published their first paper in a Scopus‐indexed journal in 2020, their research experience would be considered 5 years (2020–2024), with both 2020 and 2024 counted as full years.
Research impact metrics	MUREX and Scopus websites were used to collect data on various key impact metrics, including the H‐index, total citations, total number of research outputs, percent of international collaboration, percent of documents in top citation percentiles, percent of documents in the top 25% of journals by SCImago Journal Rank, and the field‐weighted citation impact.
H‐index	The H‐Index measures both the productivity and citation impact of a researcher’s publications. It is calculated based on the highest number of papers that have each been cited at least as many times as the index number. For example, if a researcher has an H‐index of 5, it means they have published at least 5 papers, each of which has been cited at least 5 times.
Total citations	Total citations refer to the cumulative number of times all of a researcher’s publications have been cited by other articles indexed in the Scopus database. This metric indicates the overall impact and visibility of the researcher’s work within the academic community. For example, if a researcher has 15 publications indexed in Scopus and these publications collectively receive 500 citations, the total citation count in Scopus would be 500.
Total number of research outputs	The total number of research outputs refers to the sum of all publications authored by a researcher that are indexed in the Scopus database. This metric includes various types of scholarly work, such as journal articles, conference papers, reviews, and book chapters. For instance, if a researcher has authored 20 journal articles, 5 conference papers, and 2 book chapters, the total number of research outputs in Scopus would be 27.
Percent of international collaboration	The percentage of international collaboration measures the proportion of a researcher’s publications that involve coauthors from outside their own country or region. It reflects the extent to which a researcher’s work is conducted in partnership with international colleagues. For example, if a researcher has published 10 papers and 4 of them were coauthored with researchers from other countries, the percentage of international collaboration would be 40%. This metric highlights the global nature of the researcher’s collaborations.
Percent of documents in top citation percentiles	The percentage of documents in top citation percentiles measures the proportion of a researcher’s publications that fall within the top 25% of the most cited documents globally based on the Scopus database. This metric indicates how many of the researcher’s papers are highly cited compared with all published documents worldwide. For example, if a researcher has published 20 papers and 5 of them are among the top 25% of most cited documents, the percentage of documents in the top citation percentiles would be 25%. This reflects the impact and visibility of the researcher’s work within the global academic community.
Percent of documents in the top 25% of journals by SCImago Journal Rank	The percentage of documents in the top 25% of journals by SCImago Journal Rank (SJR) measures the proportion of a researcher’s publications that are published in journals ranked in the top 25% according to SJR. SJR is a measure of journal prestige and impact based on citations received by journals. For example, if a researcher has published 30 papers and 10 of these papers appear in journals ranked in the top 25% by SJR, then the percentage of documents in the top 25% of journals by SJR is 33.3%. This metric highlights the quality and prestige of the journals where the researcher’s work is published.
The field‐weighted citation impact (FWCI)	Measures the ratio of citations received by a researcher’s publications compared to the expected global average for their specific subject field, publication type, and publication year. An FWCI of exactly 1 indicates that the research output is cited at the expected rate according to the global average. An FWCI greater than 1 signifies that the output is cited more than expected; for instance, an FWCI of 1.48 means the output is cited 48% more than the global average. Conversely, an FWCI less than 1 indicates that the output is cited less than expected relative to the global average. This metric provides insights into the relative impact of research within its field but should be used with caution, especially in areas with fewer publications, where highly cited works may disproportionately influence the result.

Faculty expertise related to SDGs (the independent variable) was identified using algorithmic classification within the MUREX system and Scopus, which categorize faculty publications based on relevance to the 17 SDGs. Each faculty member’s SDG alignment was recorded.

### 2.4. Statistical Analysis

Outcome variables (dependent variables) included the H‐index (measure of research productivity and citation impact), total citations (cumulative citations across Scopus‐indexed publications), total number of research outputs (publications indexed in Scopus), percentage of international collaboration (proportion of coauthored papers with international researchers), percentage of documents in top citation percentiles (top 25% of most cited papers), percentage of documents in the top 25% of journals (SCImago Journal Rank [SJR]) and field‐weighted citation impact (FWCI) (comparison of citation performance relative to the global average in a given discipline). Covariates and potential confounders included the academic title (Lecturer, Assistant Professor, Associate Professor, and Full Professor) and years of research experience (calculated from the year of first Scopus‐indexed publication).

Data analysis was conducted using IBM SPSS Statistics Version 20.0 and Python 3.12.4. Descriptive statistics, including frequency, percentages, means, and standard deviations (SDs), were used to describe the characteristics of nursing faculty and research impact metrics. Pearson’s correlation analysis was used as an exploratory bivariate approach to examine unadjusted relationships between faculty characteristics and research impact metrics, providing preliminary insight into variable associations prior to multivariable analysis. Multiple linear regression examined associations among these variables, reporting *R* ^2^, adjusted *R* ^2^, unstandardized and standardized beta coefficients, standard errors, *p* values, and 95% confidence intervals as applicable. Assumptions of linear regression, including normality and homoscedasticity of residuals, independence, and absence of multicollinearity, were assessed using diagnostic plots and variance inflation factors (VIFs) prior to model interpretation. Extreme Gradient Boosting (XGBoost) [12] was employed to address specific challenges in the dataset, including class imbalance, missing values, and nonlinear relationships. Its robust features, such as regularization to prevent overfitting, efficient handling of structured data, and the capability to capture complex interactions, made it well suited for this analysis. Additionally, the Synthetic Minority Oversampling Technique (SMOTE) was applied to address the class imbalance, complementing XGBoost’s ability to effectively assign weights and evaluate feature importance.

In addition to multivariable linear regression, XGBoost was employed as a supervised machine learning classification model to complement traditional regression analyses. The model was applied to classify SDG alignment categories using faculty characteristics and research impact metrics as predictors. The primary objective of this analysis was exploratory: to examine feature importance patterns and assess potential nonlinear relationships and interaction effects that may not be captured under linear regression assumptions. The model was not intended as a confirmatory validation of regression findings but rather as a complementary analytical framework. Key hyperparameters included a learning rate of 0.1, a maximum tree depth of 5, and 100 boosting iterations. These parameters were selected based on preliminary tuning to balance model complexity and generalization performance. To address class imbalance in SDG categories, SMOTE was applied exclusively within the XGBoost classification framework to address imbalance in SDG alignment categories (categorical target variable). It was not applied to continuous research impact outcomes (e.g., H‐index, citation counts, and research output), which were analyzed using regression models without synthetic resampling. The purpose of SMOTE in this context was to improve class representation during SDG classification modeling. Continuous outcome variables used in regression analyses were not subject to resampling procedures. Model performance was evaluated on an independent test set using accuracy, precision, recall, and F1‐score. Early stopping criteria were implemented to reduce the risk of overfitting.

The Strengthening the Reporting of Observational Studies in Epidemiology (STROBE) checklist [13] was used to improve the quality of reporting (Supporting Data 2).

### 2.5. Ethical Considerations

The data included in this study were publicly available through the MUREX portal and the Scopus database. Therefore, it did not require ethical approval (Project number MU‐MOU‐IRB‐NS 2025/40.1305 exemption approved by the IRB, Faculty of Nursing, Mahidol University).

## 3. Results

### 3.1. Characteristics of Nursing Faculty and Research Impact Metrics

The study sample comprised 121 nursing faculty members in Thailand. Academic titles included 26.4% Lecturers, 42.1% Assistant Professors, and 31.4% Associate Professors or above. Expertise related to the UN SDGs varied, with the majority focusing on SDG3 (Good Health and Well‐being) (100%) and SDG17 (Partnerships for Goals) (71.9%). The average number of SDGs related to each faculty member was 2.4 (SD = 1.4). Research experience averaged 7.7 years (SD = 6.0), with an H‐index of 2.2 (SD = 2.5). Faculty members had an average of 50.2 total citations (SD = 173.9) and 5.8 research outputs (SD = 7.2). On average, 25.9% of their collaborations were international (SD = 34.3%) and 10.9% of their documents were in top citation percentiles (SD = 20.5%). The average percent of documents in the top 25% of journals by SJR was 28.3% (SD = 35.4%), with a FWCI of 0.54 (SD = 0.6) (Table 2).

**TABLE 2:** Characteristics of nursing faculty and research impact.

Sample characteristics (*N* = 121)	n	%	Mean	SD
Academic title		—	—	—
Lecturer	32	26.4	—	—
Assistant Professor	51	42.1	—	—
Associate Professor or above	38	31.4	—	—
Expertise related to UN SDGs^#^	—	—	2.4	1.4
SDG1: No Poverty	< 10	—	—	—
SDG2: Zero Hunger	12	9.9	—	—
SDG3: Good Health and Well‐Being	121	100	—	—
SDG4: Quality Education	11	9.1	—	—
SDG5: Gender Equality	12	9.9	—	—
SDG6: Clean Water and Sanitation	< 10	—	—	—
SDG8: Decent Work and Economic Growth	< 10	—	—	—
SDG9: Industry, Innovation, and Infrastructure	< 10	—	—	—
SDG10: Reduced Inequalities	13	10.7	—	—
SDG11: Sustainable Cities	< 10	—	—	—
SDG13: Climate Action	< 10	—	—	—
SDG14: Life Below Water	< 10	—	—	—
SDG16: Peace, Justice, and Strong Institutions	18	14.9	—	—
SDG17: Partnership for the Goals	87	71.9		
Year of research experience^∗^	—	—	7.7	6.0
H‐index	—	—	2.2	2.5
Total citation	—	—	50.2	173.9
Total number of research outputs	—	—	5.8	7.2
Percent of international collaboration	—	—	25.9	34.3
Percent of documents in top citation percentiles	—	—	10.9	20.5
Percent of documents in the top 25% of journals by SCImago Journal Rank^∗∗^	—	—	28.3	35.4
Field‐weighted citation impact	—	—	0.54	0.6

*Note:* There is no expertise related to SDG7, SDG12, and SDG15; therefore, no data are available.

Abbreviations: UN = United Nations, SDGs = Sustainable Development Goals.

^#^The number (*n*) may be greater than the total sample size, as one sample can contribute more than one SDG.

^∗^The first year counting from the first research paper published in a Scopus‐indexed journal.

^∗∗^The SCImago Journal Rank indicator measures the prestige of scholarly journals by considering both the number of citations a journal receives and the prestige of the journals from which those citations originate [14].

### 3.2. Pearson’s Correlation Between Characteristics of Nursing Faculty and Research Impact Metrics

Pearson’s correlation matrix for the characteristics of nursing faculty and research impact metrics highlighted several significant relationships (Table 3). Academic titles showed a positive correlation with various research impact metrics, including expertise related to UN SDGs (*r* = 0.27, *p* < 0.01), years of research experience (*r* = 0.49, *p* < 0.01), H‐index (*r* = 0.31, *p* < 0.01), total citations (*r* = 0.21, *p* < 0.01), and the total number of research outputs (*r* = 0.34, *p* < 0.01). This indicates that faculty members with higher academic titles tend to achieve higher research impact across these metrics. Similarly, expertise in UN SDGs was significantly associated with years of research experience (*r* = 0.42, *p* < 0.01), H‐index (*r* = 0.56, *p* < 0.01), total citations (*r* = 0.42, *p* < 0.01), and the total number of research outputs (*r* = 0.63, *p* < 0.01). This indicates that faculty members with more expertise in UN SDGs tend to achieve higher research impact across these metrics.

**TABLE 3:** Pearson’s correlation matrix for characteristics of nursing faculty and research impact metrics.

Sample characteristics (*n* = 121)	1	2	3	4	5	6	7	8	9	10
1. Academic title	1	—	—	—	—	—	—	—	—	—
2. Expertise related to UN SDGs	0.27^∗∗^	1	—	—	—	—	—	—	—	—
3. Year of research experience	0.49^∗∗^	0.42^∗∗^	1	—	—	—	—	—	—	—
4. H‐index	0.31^∗∗^	0.56^∗∗^	0.65^∗∗^	1	—	—	—	—	—	—
5. Total citation	0.21^∗^	0.42^∗∗^	0.52^∗∗^	0.82^∗∗^	1	—	—	—	—	—
6. Total number of research outputs	0.34^∗∗^	0.63^∗∗^	0.61^∗∗^	0.92^∗∗^	0.77^∗∗^	1	—	—	—	—
7. Percent of international collaboration	−0.02	0.13	0.11	0.23^∗∗^	0.16	0.20^∗^	1	—	—	—
8. Percent of documents in top citation percentiles	0.05	0.07	0.07	0.33^∗∗^	0.17	0.21^∗^	0.11	1	—	—
9. Percent of documents in the top 25% of journals by SCImago Journal Rank	−0.15	0.02	−0.12	0.06	0.02	−0.01	0.06	0.21^∗^	1	—
10. Field‐weighted citation impact	−0.06	0.15	0.14	0.47^∗∗^	0.31^∗∗^	0.31^∗∗^	0.20^∗^	0.76^∗∗^	0.32^∗∗^	1

^∗^Correlation is significant at *p* value < 0.05 level (2‐tailed).

^∗∗^Correlation is significant at *p* value < 0.01 level (2‐tailed).

The number of years of research experience was positively correlated with the H‐index (*r* = 0.65, *p* < 0.01), total citations (*r* = 0.52, *p* < 0.01), and total number of research outputs (*r* = 0.61, *p* < 0.01), highlighting that increased research experience is associated with greater research impact. Other notable correlations include a strong relationship between the H‐index and total citations (*r* = 0.82, *p* < 0.01) and between the H‐index and the total number of research outputs (*r* = 0.92, *p* < 0.01). This suggests that higher H‐index scores are closely related to increased total citations and research outputs. The percentage of documents in top citation percentiles was correlated with FWCI (*r* = 0.76, *p* < 0.01), indicating that documents with higher citation percentiles are also associated with greater impact.

Conversely, the percentage of international collaboration had weaker correlations with research impact metrics (e.g., *r* = 0.23 with H‐index, *p* < 0.01) and the percentage of documents in the top 25% of journals by SJR had weak to moderate correlations with other metrics. Overall, these findings highlight the significant relationships between expertise in UN SDGs, years of research experience, and various research impact metrics, while also emphasizing less pronounced associations for some other metrics.

### 3.3. Associations Between Characteristics of Nursing Faculty and Research Impact Metrics

**FIGURE 1:**
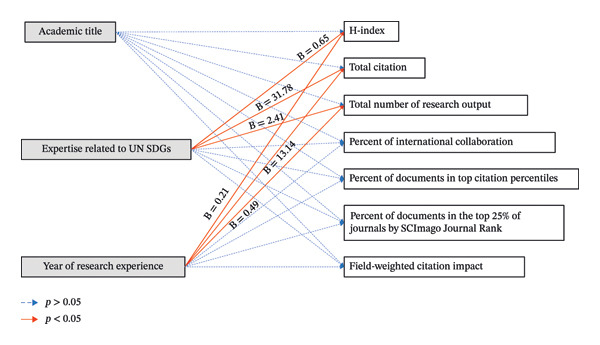
Association between characteristics of nursing faculty and research impact metrics. *Notes*. Statistically significant at *p* value < 0.05, *B* = unstandardized coefficients; dependent variable: H‐index: a significant equation was found (*F* [3, 117] = 42.4, *p* < 0.001) with *R* ^2^ = 0.52 and adjusted *R* ^2^ = 0.51. Dependent variable: total citation: a significant equation was found (*F* [3, 117] = 18.4, *p* < 0.001) with *R* ^2^ = 0.32 and adjusted *R* ^2^ = 0.3. Dependent variable: total number of research outputs: a significant equation was found (*F* [3, 117] = 46.1, *p* < 0.001) with *R* ^2^ = 0.54 and adjusted *R* ^2^ = 0.53.

Multiple regression analyses (Figure 1) showed that expertise related to UN SDGs was a significant predictor of the H‐index (*β* = 0.65, *p* < 0.001), total citations (*β* = 31.78, *p* = 0.004), and total number of research outputs (*β* = 2.41, *p* < 0.001). Additionally, years of research experience were a significant predictor of the H‐index (*β* = 0.21, *p* < 0.001), total citations (*β* = 13.14, *p* < 0.001), and total number of research outputs (*β* = 0.49, *p* < 0.001). Other associations were not significant. The full results can be found in Supporting Data 3.

### 3.4. Key Faculty Characteristics and Expertise Areas Influencing Research Impact Metrics

#### 3.4.1. Data Preparation for XGBoost

**FIGURE 2:**
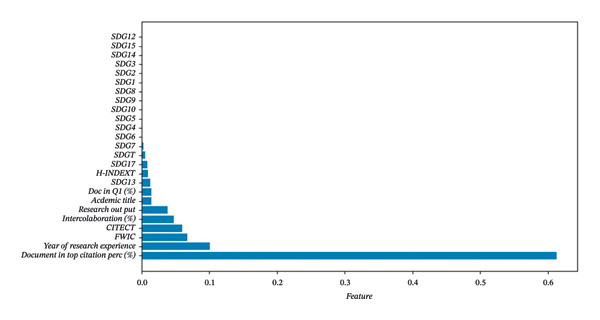
The related parameter for XGBoost: addressing class impact in SDG indicators. *Note*. SDGs: Sustainable Development Goals; CITECT: total citations; Doc in Q1(%): percentage of documents in the top 25% of journals by SCImago Journal Rank; FWCI: field‐weighted citation impact.

The dataset comprised both categorical and numerical variables (as described in “Measures”) relevant to analyzing the research impact metrics of faculty members. The dataset highlighted a class imbalance among the SDG indicators, with some goals, such as SDG13 (Climate Action) and SDG17 (Partnerships for the Goals), being more frequently represented in research alignment, while others, such as SDG12 (Responsible Consumption and Production) and SDG 15 (Life on Land), have limited representation. This imbalance has implications for the performance of machine learning models, as shown in Figure 2. In view of this class imbalance, resampling techniques or class‐weighting methods are indicated to ensure that the models generate reliable insights. The preprocessed dataset was then split into training and testing sets using an 80–20 ratio to allow for model training and independent evaluation. Stratified sampling was employed to maintain the class distribution across both sets, ensuring a fair evaluation of the model on the test data.

#### 3.4.2. Confusion Matrix of the XGBoost Model

**FIGURE 3:**
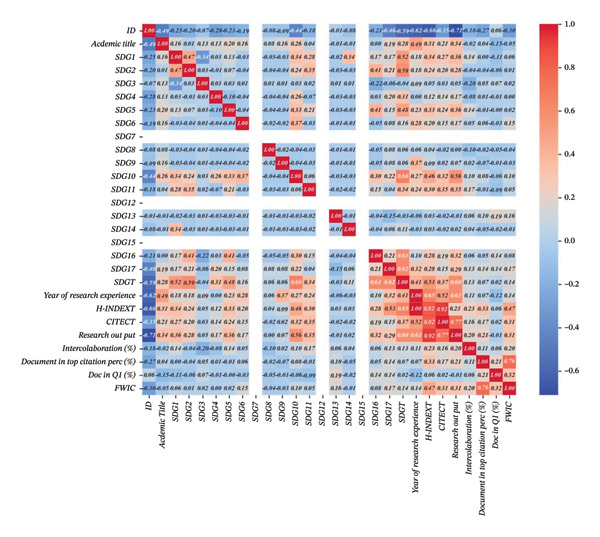
The confusion matrix of the XGBoost model. *Note*. ID: Study ID; SDGs: Sustainable Development Goals; CITECT: total citations; Doc in Q1(%): percentage of documents in the top 25% of journals by SCImago journal rank; FWCI: field‐weighted citation impact.

The classification performance of the XGBoost model was evaluated on the test dataset, focusing on both overall metrics and class‐specific performance. The results indicate that the proposed approach effectively classifies the data, particularly in addressing challenges posed by class imbalance and complex feature‐label relationships (Figure 3). The model achieved an overall accuracy of 98.4%, demonstrating its capability to correctly classify the majority of instances. Confusion metrics were calculated to further assess the model’s reliability. The weighted average precision across all classes was 98.0%, reflecting the model’s ability to minimize false positives. The overall recall was 94.8%, indicating robust detection of instances for each class, including minority classes, following the application of SMOTE (Supporting Data 4).

Class‐specific performance metrics provided additional insights into the model’s ability to distinguish between different categories. For each class, precision and recall were analyzed, and for the majority of classes, these metrics were consistently high. A detailed analysis of the confusion matrix revealed that the model effectively reduced misclassifications between similar classes. However, some overlap in predictions persisted for certain closely related categories, highlighting potential areas for further improvement, such as feature engineering or hyperparameter tuning.

#### 3.4.3. Model Performance

XGBoost demonstrated superior performance compared with logistic regression and random forest models, particularly after the application of SMOTE (Table 4). The improvement was most evident in recall, indicating enhanced detection of minority SDG classes. These findings suggest that nonlinear ensemble methods would be better at capturing complex interactions among faculty characteristics and research impact metrics under imbalanced conditions.

**TABLE 4:** Comparative classification performance of logistic regression, random forest, and XGBoost models.

Model	Accuracy (%)	Precision	Recall	F1‐score
Logistic regression	82.3	0.78	0.74	0.76
Random forest	91.2	0.89	0.86	0.87
XGBoost (no SMOTE)	93.7	0.91	0.88	0.89
XGBoost + SMOTE	98.4	0.98	0.95	0.96

The primary purpose of the XGBoost analysis was to identify influential predictors and to evaluate classification performance under nonlinear modeling assumptions rather than to establish causal relationships or replace regression analysis. To examine model performance, violin plots were generated to visualize the distribution of key evaluation metrics (accuracy, precision, and recall) across SDG classes before and after SMOTE and XGBoost application (Figures 4(a) and 4(b)). Metrics for the majority classes were concentrated in the higher range, indicating stable classification performance. Minority classes demonstrated greater variability in the pre‐SMOTE condition (Figure 4(a)), reflecting residual challenges under imbalanced class distributions. Following SMOTE implementation, performance distributions became more concentrated in the higher range (Figure 4(b)), particularly for recall, indicating improved minority class detection. These visualizations provide insight into class‐specific performance patterns rather than serving as confirmatory evidence of model superiority.

FIGURE 4Distribution of SDS violin plots before (a) and after (b) applying SMOTE and XGBoost algorithms.

**Figure:**
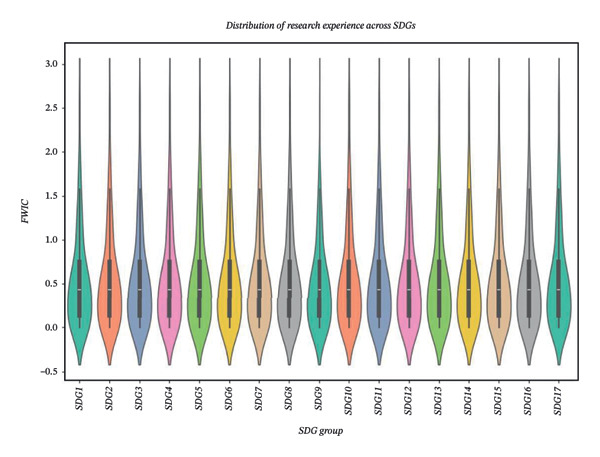
(a)

**Figure:**
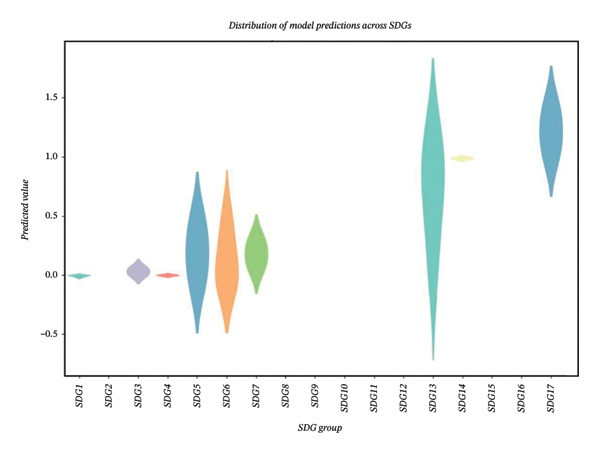
(b)

Comparative analyses indicated improved classification performance following SMOTE application (Table 4). XGBoost demonstrated higher overall evaluation metrics relative to baseline models; however, given the modest sample size, these findings should be interpreted as exploratory. The model’s ensemble structure allows flexibility in capturing potential nonlinear relationships and interaction effects within structured data. Importantly, the machine learning analysis was conducted as a complementary exploratory approach. Whereas regression models provide interpretable effect estimates under linear assumptions, XGBoost facilitated the examination of variable importance rankings and potential interaction structures. Consistency in influential predictors across modeling approaches supports the robustness of observed associations although all findings remain associative rather than causal.

We observed a strong positive correlation between the percentage of documents in the top citation percentiles and year of research experience, suggesting mutual dependence between these indicators of research impact. The percentage of international collaboration showed a moderate positive correlation with the percentage of documents in top citation percentiles, suggesting that collaborative research efforts may contribute to the production of higher‐quality publications. When examined in the context of SDG indicators, the highest values for the percentage of documents in top citation percentiles were frequently associated with specific goals, such as SDG 13 (Climate Action) (Figure 4(b)).

## 4. Discussion

Overall, this study shows that nursing faculty expertise in SDGs is significantly associated with research impact, with engagement across multiple SDGs, particularly SDG3 and SDG17, linked to higher H‐index values, citation counts, and research output. Research experience remained a strong determinant of academic impact, reflecting cumulative scholarly productivity over time. In addition, the inclusion of XGBoost was not intended to replace traditional regression analysis but to complement it. Whereas regression models provide interpretable effect estimates and inferential statistics, XGBoost enables exploration of nonlinear interactions and improves predictive classification performance under class imbalance. The comparative analysis demonstrated that ensemble‐based approaches achieved higher recall and overall classification accuracy, supporting their utility in an exploratory modeling context.

Specifically, XGBoost highlighted the combined influence of years of experience, breadth of SDG expertise, and international collaboration, revealing interaction patterns that linear regression models did not fully capture. Faculty aligned with SDG13 showed a higher proportion of top‐cited publications; however, this association should be interpreted cautiously, as it may reflect broader global citation and policy attention trends rather than nursing‐specific research dynamics. In contrast, SDG12 and SDG15 were underrepresented, indicating opportunities for nursing research to engage more broadly with sustainability domains beyond traditional health‐focused priorities.

Along with expertise in the SDGs and years of research experience predicting the H‐index, high‐impact research in nursing and health was particularly linked to SDG13, which focuses on climate action. This observation aligns with previous studies demonstrating that climate‐related research tends to receive higher citation rates on average than many other research areas [15–17]. Notably, whereas SDG‐related research constitutes less than 30% of all papers indexed in Scopus, it accounts for approximately 66% of research cited in policy documents, and SDG‐related studies are cited almost five times more frequently in policy contexts than non‐SDG research [18]. Research relevant to SDG3 represents nearly 40% of all publications, with approximately 30% cited in policy documents [18], suggesting that citation advantages may be driven by policy relevance and global research visibility rather than disciplinary specificity alone. Climate change and health‐related diseases are also among the fastest‐growing publication areas and together comprise more than half of the most highly cited articles [16, 17]. Increased accessibility, heightened public visibility, and policy relevance likely contribute to these citation patterns.

Journal characteristics may also influence citation impact [16, 17]. However, due to the lack of detailed journal‐level data, firm conclusions regarding journal effects cannot be drawn. Prior evidence suggests that well‐designed research aligned with SDGs—particularly SDG3 and SDG13—and published in high‐impact, widely accessible journals is more likely to achieve greater citation visibility [19–21]. Accordingly, the observed prominence of SDG13 should be viewed as indicative of broader global research and policy agendas rather than as definitive evidence of SDG‐specific effects within nursing scholarship.

The study further indicates that broader expertise across UN SDGs among nursing faculty members significantly predicts academic impact, as reflected in the H‐index, total citations, and research output. This underscores the role of SDG‐aligned research in shaping scholarly productivity and influence within nursing and aligns with prior evidence that research addressing public health and sustainability attracts greater attention, funding, and interdisciplinary collaboration [22, 23]. On average, nursing faculty members demonstrated expertise in 2.4 SDGs, with SDG3 and SDG17 being the most common. Expanding engagement across a wider range of SDGs remains important, as nursing can contribute meaningfully to advancing multiple sustainability goals, including SDG5 (Gender Equality) and SDG10 (Reducing Inequalities) [24]. Furthermore, as the largest group of healthcare professionals worldwide, nurses have the potential to influence policy and advocate for climate action (SDG13) [25]. Institutional support for interdisciplinary collaboration and structured SDG‐oriented research initiatives may further strengthen this alignment.

Finally, years of research experience consistently predicted the H‐index, total citations, and research output. Greater research experience contributes to enhanced methodological expertise, scientific credibility, and leadership capacity, which facilitate publication in high‐impact journals and sustained scholarly influence [26]. Total citations similarly accumulate over time, reflecting longer exposure of published work within academic networks. Previous studies have shown that senior faculty members tend to have higher citation counts than junior colleagues due to extended publication histories and broader collaborative opportunities [27]. Nevertheless, external forces such as shifting research priorities and public health crises may temporarily alter citation trajectories. For example, during the COVID‐19 pandemic, accelerated publication processes and heightened attention to pandemic‐related research may have influenced citation patterns across disciplines [28].

### 4.1. Study Limitations

The principal limitations of this study are related to its cross‐sectional design, which captures associations at a single time point and limits inference regarding causality or long‐term trends. As faculty expertise and research impact may evolve due to institutional policies, funding mechanisms, or shifting research priorities, longitudinal designs are needed to provide deeper insights. In addition, the study focused on a single institution (Mahidol University, Thailand), which may limit generalizability. Future studies should include multi‐institutional and international samples to validate these findings.

Another key limitation is that data were drawn from publicly available databases (MUREX and Scopus), which may not fully capture faculty expertise or total research contributions. The algorithmic classification of SDG expertise may not fully reflect actual faculty research focus, and citation‐based metrics (H‐index and citation counts) do not capture research quality, societal impact, or policy influence. Future studies should consider incorporating alternative indicators, such as Altmetrics and policy impact measures.

Furthermore, while XGBoost improved predictive performance relative to traditional regression, machine learning models remain limited in interpretability and do not establish causation. In addition, although SMOTE was used to address imbalance in SDG representation and enhance model performance, it introduces synthetic data that may not fully reflect real‐world distributions. Alternative approaches, such as cost‐sensitive learning, should be explored in future research.

Crucially, most faculty expertise was concentrated in SDG3 (Health) and SDG17 (Partnerships), whereas SDG12 (Responsible Consumption) and SDG15 (Life on Land) were minimally represented. This pattern suggests a disciplinary concentration within nursing research and highlights the need to expand engagement with broader sustainability challenges.

Finally, as research impact metrics and SDG alignment become increasingly integrated into faculty evaluation systems, ethical considerations warrant attention. There is a risk that SDG alignment could be interpreted as a performance requirement rather than a supportive framework. Institutions should, therefore, ensure that the use of bibliometric indicators and SDG‐related metrics remains constructive, transparent, and nonpunitive.

### 4.2. Future Research Directions and Implementation

The findings of this study underscore the role of nursing faculty expertise in the UN SDGs and research experience in shaping academic impact, as reflected in the H‐index, total citations, and research output. Future research should prioritize longitudinal study designs to examine how SDG expertise and research impact evolve over time, enabling stronger assessment of temporal dynamics and potential causal relationships.

To strengthen external validity, subsequent studies should incorporate multi‐institutional and multicountry samples, including nursing faculty from diverse academic settings within Thailand and internationally. Such approaches would allow comparison across institutional, cultural, and policy contexts and provide a broader understanding of SDG‐aligned nursing scholarship.

Given the interdisciplinary scope of the SDGs, future investigations should also examine how cross‐disciplinary collaboration influences research visibility, citation performance, and policy engagement. Collaboration across nursing, public health, environmental science, social sciences, and related fields may clarify mechanisms through which SDG‐aligned research achieves enhanced academic and societal impact.

Although the integration of SDG principles into nursing education remains relevant, detailed curricular implementation extends beyond the scope of the present study and warrants dedicated educational research.

## 5. Conclusion

The findings of this study highlight the crucial role of nursing faculty expertise in SDGs and their research experience in shaping academic impact, as evidenced by key metrics including the H‐index, total citations, and research output. These results emphasize the importance of aligning nursing research with SDGs to enhance its quality, visibility, and global influence. Additionally, this study revealed the increasing prominence of SDG 13 (Climate Action) in nursing research, with this area consistently associated with the highest percentage of top‐cited documents. This trend suggests that climate action–focused nursing research is gaining considerable attention and is recognized as highly impactful within the academic community. By embedding SDG‐related competencies into nursing education and research, the profession can play a pivotal role in advancing the UN SDG agenda and addressing pressing global challenges.

## Funding

No funding was received for this study. Mahidol University, Thailand, supported the Article Processing Charge. Imperial College London is grateful for funding from the National Institute for Health & Care Research (NIHR) Applied Research Collaboration NorthWest London.

## Disclosure

The views expressed in this article are those of the authors and not necessarily those of the NIHR or Department of Health and Social Care.

## Conflicts of Interest

The authors declare no conflicts of interest.

## Supporting Information

Additional supporting information can be found online in the Supporting Information section.

Supporting Data 1–4 related to this article can be found in the online version.

## Supporting information


**Supporting Information 1** Supporting Data 1. Nursing Research Expertise Aligned with the UN Sustainable Development Goals.

**Supporting Information 2** Supporting Data 2. STROBE Statement—Checklist of items that should be included in reports of cross‐sectional studies.

**Supporting Information 3** Supporting Data 3. The Association Among Characteristics of the Sample.

**Supporting Information 4** Supporting Data 4. The Confusion Matrix.

## Data Availability

The data that support the findings of this study are openly available in MUREX Portal and the Scopus databases at https://murex.mahidol.ac.th/, and https://www.scopus.com/pages/home?display=basic&zone=header&origin=AuthorProfile#basic, reference number [10, 11].
