# Supplementary material for: Associations Between Nursing Faculty Expertise in the United Nations Sustainable Development Goals and Research Impact Metrics: A Cross‐Sectional Study
Source: J Nurs Manag. 2026 Apr 7;2026:9740644. doi: 10.1155/jonm/9740644 (PMC13054229; doi:10.1155/jonm/9740644)
Supplement: Supplementary file 1 — Supporting Information 1 Supporting Data 1. Nursing Research Expertise Aligned with the UN Sustainable Development Goals. [file JONM-2026-9740644-s004.docx]

**Supplementary Data 1**

**Nursing Research Expertise Aligned with the UN Sustainable Development Goals Framework**

1. No Poverty: Eradicate extreme poverty and reduce poverty rates globally by promoting economic stability and social protection.

**Nursing research:** Address health disparities and access to care for impoverished populations.

2. Zero Hunger: Achieve food security and improved nutrition for all by supporting sustainable agriculture and equitable food distribution.

**Nursing research:** Focus on nutrition-related health issues and interventions for vulnerable groups.

3. Good Health and Well-Being: Ensure healthy lives and well-being at all ages through improved healthcare access, disease prevention, and health promotion.

**Nursing research:** Central to advancing healthcare practices, patient outcomes, and public health strategies.

4. Quality Education: Promote inclusive and equitable quality education and lifelong learning opportunities.

**Nursing research:** Contribute to the development of effective educational programs and training for future healthcare professionals.

5. Gender Equality: Achieve gender equality and empower women and girls by addressing disparities and promoting equal opportunities.

**Nursing research:** Explore gender-specific health issues and interventions to support gender equality in healthcare.

6. Clean Water and Sanitation: Ensure access to clean water and sanitation for all, improving health and hygiene standards.

**Nursing research:** Investigate the impact of water and sanitation on health and develop solutions for better health outcomes.

7. Affordable and Clean Energy: Provide access to sustainable and modern energy sources, reducing energy poverty and environmental impact.

**Nursing research:** Assess the health impacts of energy access and promote energy-efficient healthcare practices.

8. Decent Work and Economic Growth: Promote sustainable economic growth and decent work opportunities, ensuring fair labor practices and productive employment.

**Nursing research:** Address occupational health and safety issues within the healthcare sector.

9. Industry, Innovation, and Infrastructure: Build resilient infrastructure and foster innovation to support sustainable industrialization.

**Nursing research:** Contribute to technological advancements and infrastructure improvements in healthcare settings.

10. Reduced Inequalities: Reduce inequality within and among countries by promoting inclusive growth and social equity.

**Nursing research:** Focus on reducing health disparities and improving access to care for marginalized populations.

11. Sustainable Cities and Communities: Create safe, resilient, and sustainable urban environments.

**Nursing research:** Explore the impact of urbanization on health and develop strategies for improving community health and resilience.

12. Responsible Consumption and Production: Promote sustainable consumption and production patterns to minimize environmental impact.

**Nursing research:** Address the environmental effects of healthcare practices and advocate for sustainable practices in the industry.

13. Climate Action: Take urgent action to combat climate change and its effects on health and the environment.

**Nursing research:** Investigate the health impacts of climate change and develop adaptation strategies for healthcare systems.

14. Life Below Water: Conserve and sustainably use marine resources and protect marine ecosystems.

**Nursing research:** Explore the health effects of marine pollution and advocate for policies to safeguard aquatic environments**.**

15. Life on Land: Protect and restore terrestrial ecosystems, manage forests sustainably, and combat land degradation.

**Nursing research:** Examine the health impacts of land degradation and promote conservation efforts.

16. Peace, Justice, and Strong Institutions: Promote peaceful societies, access to justice, and effective institutions.

**Nursing research:** Address the health impacts of conflict and violence and support policies for justice and health equity.

17. Partnerships for the Goals: Strengthen global partnerships and collaborative efforts to achieve the SDGs.

**Nursing research:** Contribute to international collaborations and knowledge sharing to address global health challenges.

**Reference**

United Nations. The UN Sustainable Development Goals. United Nations, New York, 2015. Available at (accessed 25 August 2024): <http://www.un.org/sustainabledevelopment/summit/>.
