# Supplementary material for: Associations Between Nursing Faculty Expertise in the United Nations Sustainable Development Goals and Research Impact Metrics: A Cross‐Sectional Study
Source: J Nurs Manag. 2026 Apr 7;2026:9740644. doi: 10.1155/jonm/9740644 (PMC13054229; doi:10.1155/jonm/9740644)
Supplement: Supplementary file 3 — Supporting Information 3 Supporting Data 3. The Association Among Characteristics of the Sample. [file JONM-2026-9740644-s002.docx]

**Supplementary Data 3**

**The Association Among Characteristics of the Sample**

| **Predictors** | **Unstandardized Coefficients** | | **p-value** | **95% C.I.** | |
| --- | --- | --- | --- | --- | --- |
|  | **B** | **Std. Error** |  | **Lower Bound** | **Upper Bound** |
| **Dependent Variable: H-Index** | | | | | |
| Academic Title | -0.128 | 0.238 | 0.592 | -0.599 | 0.344 |
| Expertise related to UN SDGs | 0.647 | 0.129 | 0.000 | 0.391 | 0.903 |
| Year of Research Experience | 0.213 | 0.032 | 0.000 | 0.149 | 0.277 |
| **Dependent Variable: Total citation** | | | | | |
| Academic Title | -17.508 | 19.984 | .383 | -57.085 | 22.069 |
| Expertise related to UN SDGs | 31.770 | 10.839 | 0.004 | 10.303 | 53.236 |
| Year of Research Experience | 13.135 | 2.703 | 0.000 | 7.781 | 18.489 |
| **Dependent Variable: Total number of research outputs** | | | | | |
| Academic Title | 0.161 | 0.679 | 0.813 | -1.184 | 1.505 |
| Expertise related to UN SDGs | 2.409 | 0.368 | 0.000 | 1.680 | 3.138 |
| Year of Research Experience | 0.493 | 0.092 | 0.000 | 0.311 | 0.675 |
| **Dependent Variable: Percent of international collaboration** | | | | | |
| Academic Title | -4.709 | 4.714 | 0.320 | -14.046 | 4.628 |
| Expertise related to UN SDGs | 2.857 | 2.557 | 0.266 | -2.207 | 7.921 |
| Year of Research Experience | 0.662 | 0.638 | 0.301 | -0.601 | 1.926 |
| **Dependent Variable: Percent of documents in top citation percentiles** | | | | | |
| Academic Title | 0.280 | 2.852 | 0.922 | -5.368 | 5.928 |
| Expertise related to UN SDGs | 0.798 | 1.547 | 0.607 | -2.265 | 3.862 |
| Year of Research Experience | 0.142 | 0.386 | 0.713 | -0.622 | 0.906 |
| **Dependent Variable: Percent of documents in the top 25% of journals by SCImago Journal Rank** | | | | | |
| Academic Title | -5.843 | 4.856 | 0.231 | -15.459 | 3.774 |
| Expertise related to UN SDGs | 2.480 | 2.634 | 0.348 | -2.736 | 7.696 |
| Year of Research Experience | -0.568 | 0.657 | 0.389 | -1.869 | 0.732 |
| **Dependent Variable: Field-Weighted Citation Impact** | | | | | |
| Academic Title | -0.122 | 0.074 | 0.104 | -0.270 | 0.025 |
| Expertise related to UN SDGs | 0.048 | 0.040 | 0.236 | -0.032 | 0.128 |
| Year of Research Experience | 0.016 | 0.010 | 0.123 | -0.004 | 0.036 |

***Note.*** Statistically significant at p < 0.05.
