# Supplementary material for: Associations Between Nursing Faculty Expertise in the United Nations Sustainable Development Goals and Research Impact Metrics: A Cross‐Sectional Study
Source: J Nurs Manag. 2026 Apr 7;2026:9740644. doi: 10.1155/jonm/9740644 (PMC13054229; doi:10.1155/jonm/9740644)
Supplement: Supplementary file 4 — Supporting Information 4 Supporting Data 4. The Confusion Matrix. [file JONM-2026-9740644-s003.docx]

**Supplementary Data 4**

**The Confusion Matrix**

|  | **Predicted Positive** | **Predicted Negative** |
| --- | --- | --- |
| **Actual Positive** | True Positive (TP) | False Negative (FN) |
| **Actual Negative** | False Positive (FP) | True Negative (TN) |

**Note:** True Positive (TP) is the number of instances where the model correctly predicted the positive class. True Negative (TN) is the number of instances where the model correctly predicted the negative class. False Positive (FP) is the number of instances where the model incorrectly predicted the positive class (also known as a Type I error). False Negative (FN) is the number of instances where the model incorrectly predicted the negative class (also known as a Type II error).

**Formula for Accuracy, Precision, and Recall Calculation**

1. $Accuracy= \frac{(TPs + TNs)}{(TPs+TNs+FPs + FNs)}$
2. $Precision= \frac{\mathrm{TPs}}{(TPs+FPs)}$
3. $Recall = \frac{\mathrm{TPs}}{(TPs+FNs)}$
